# Neuroprotective Effects of Agri-Food By-Products Rich in Phenolic Compounds

**DOI:** 10.3390/nu15020449

**Published:** 2023-01-14

**Authors:** Alejandro Rojas-García, Álvaro Fernández-Ochoa, María de la Luz Cádiz-Gurrea, David Arráez-Román, Antonio Segura-Carretero

**Affiliations:** Department of Analytical Chemistry, University of Granada, 18071 Granada, Spain

**Keywords:** neurodegeneration, plant by-products, phenolic compounds, neuroprotection, oxidative stress, protein aggregation, neuroinflammation, mitochondrial dysfunction, AChE

## Abstract

Neurodegenerative diseases are known for their wide range of harmful conditions related to progressive cell damage, nervous system connections and neuronal death. These pathologies promote the loss of essential motor and cognitive functions, such as mobility, learning and sensation. Neurodegeneration affects millions of people worldwide, and no integral cure has been created yet. Here, bioactive compounds have been proven to exert numerous beneficial effects due to their remarkable bioactivity, so they could be considered as great options for the development of new neuroprotective strategies. Phenolic bioactives have been reported to be found in edible part of plants; however, over the last years, a large amount of research has focused on the phenolic richness that plant by-products possess, which sometimes even exceeds the content in the pulp. Thus, their possible application as an emergent neuroprotective technique could also be considered as an optimal strategy to revalorize these agricultural residues (those originated from plant processing). This review aims to summarize main triggers of neurodegeneration, revise the state of the art in plant extracts and their role in avoiding neurodegeneration and discuss how their main phenolic compounds could exert their neuroprotective effects. For this purpose, a diverse search of studies has been conducted, gathering a large number of papers where by-products were used as strong sources of phenolic compounds for their neuroprotective properties. Finally, although a lack of investigation is quite remarkable and greatly limits the use of these compounds, phenolics remain attractive for research into new multifactorial anti-neurodegenerative nutraceuticals.

## 1. Introduction

The neurodegeneration process is based on the continuous dysfunction of nerve structures and neuronal loss, progressively causing a detrimental effect on cognitive and motor-related abilities, such as memory, learning, decision making, balance, movement, talking, breathing and heart function [1]. Among age-related diseases, chronic neurodegeneration prevalence has increasingly grown over the last decades, being one of the most current urgent health concerns in society [2].

The most common neurodegenerative disorders are Alzheimer’s disease (AD) and Parkinson’s disease (PD). In AD, the inhibition of correct neuronal function hinders one’s communication, which finally leads to memory loss, cognitive decline and dementia. This condition affects about 1 in 9 people (10.7%) aged 65 and older, and about 11 in 10000 people (0.11%) under 65 in the U. S. [3]. It was estimated that around 58.66 million people suffered from AD in 2020 worldwide, a number expected to triplicate by 2050 [4]. PD symptoms are more related to motor function diminishing, such as resting tremor, postural imbalance, bradykinesia and muscular rigidity [5,6]. In 2019, it was estimated that more than 10 million people worldwide lived with this disease [7].

The etiology of AD, PD and other neurological pathologies still has not yet been fully understood, partly due to the arduous access to the human brain for its investigation [8]. Therefore, there are currently no effective treatments for a complete prevention, only allowing for the preclusion of its progression to a certain extent with medications whose long-term use can lead to hazardous side effects [8]. No clinical study has been able to prove the full prevention of disease progression [9,10].

For this reason, some emergent neuroprotective strategies are focusing on treating neurodegeneration with new techniques and technologies, such as combinatorial therapies, diet interventions and/or personalized genomic studies [11,12]. The promotion of new natural antioxidants to target brain disorders has resulted in unsuccessful clinical outcomes [13]. Nevertheless, the use of extracts rich in bioactive compounds, which apart from exerting a noteworthy antioxidant activity can interact with signaling cascades and molecular pathways in human physiology, is a potential neuroprotective research line [12,13]. Moreover, science has evolved and now allows the development of a variety of experimental models that try to mimic desired human aspects as far as possible using animals and in vitro culture cells [8]. Animal models such as *Drosophila melanogaster*, *Danio rerio*, *Rattus norvegicus* or *Caenorhabditis elegans* are commonly used due to their rapid development, small sizes and high homology to humans [8,10,14,15].

Plant-derived bioactive compounds (phytochemicals) have been highlighted as great therapeutical options for their preventive and biological potential in several human disorders [16]. In the brain, phytochemicals show this therapeutical profile by exerting antioxidant and anti-inflammatory activities, as well as different protective mechanisms which may make them potentially useful in counteracting neurodegeneration [17]. Specifically, phenolic compounds, which represent the most widely distributed bioactives in the human diet, are considered a promising source of compounds for the treatment of age-related cognitive decline and the risk of developing neurodegeneration due to their antioxidant and anti-inflammatory properties. However, limitations related to their bioavailability and permeability through the blood–brain barrier (BBB) must be considered in the development of therapeutic applications with these compounds [1,18].

Previous studies have demonstrated the neuroprotective potential of specific phenolic compounds, such as Roy et al. (2019), which stated that epigallocatechin gallate from green tea was able to suppress fibrillation and protein aggregation in PD [19]. Moreover, dos Santos et al. (2021) extracted piceatannol stilbene from *Passiflora edulis* and demonstrated its in vitro activity diminishing choline degradation and neuroinflammation [20]. On the other hand, the direct application of fruit and vegetable extracts have also shown bioactive potential for neuroprotection and brain health [21,22]. In both cases, fruit by-products were used as phenolic sources, showing interesting in vivo neuroprotective effects. The qualitative determination of vegetal matrices allows authors to determine which compounds exerted these benefits. For Maurya (2019), *Swietenia macrophyla* seed contained high quantity of alkaloids, tannins, terpenoids and flavonoids [21]. In the case of Ortega-Arellano et al. (2019), avocado peel showed high amounts of B-type procyanidins (dimers, trimers and tetramers), flavanols monomers and chlorogenic acids, which were responsible for the neuroprotective effect on *Drosophila melanogaster* species [22]. In this regard, the food industry has focused in recent years on reusing by-products as a method of promoting bio-circular economy and reducing contamination, since food waste generates 8% of the global greenhouse gas emissions [23]. Therefore, the use of phenolics would not only provide an alternative way to prevent or treat neuropathologies, but also would establish an important strategy for the revalorization of agri-food waste. Until now, the neuroprotective role of agri-food by-product extracts has been scarcely studied. In this context, a comprehensive review of the state of the art in the application of by-products as neuroprotective agents against Alzheimer’s and Parkinson’s diseases is carried out in order to highlight the importance of their revalorization and to find new treatments to alleviate these neurodegenerative effects.

## 2. Materials and Methods

### 2.1. Search Strategy

All the scientific information required to complete this review has been gathered from the previous literature contained in Web of Science (WoS) Core Collection database.

**Figure 1:**
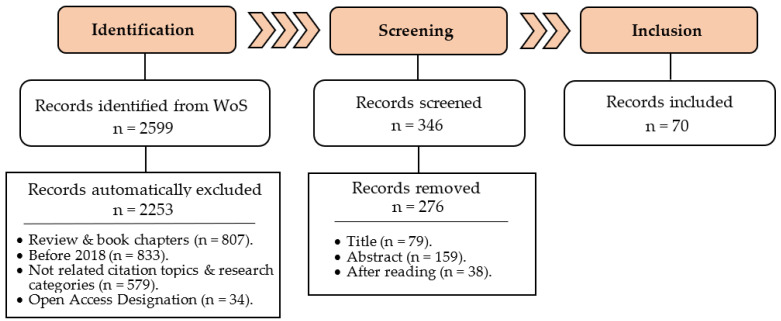
Flowchart summarizing the literature selection process according to PRISMA methodology.

The core search was performed up to the 30th of September. PRISMA methodology (Figure 1) was applied using the following keywords: (by-product or seed or peel or fruit or leaf or leaves) AND (phenolic or bioactive or bio-active or polyphenol or flavonoid or phytochemical or gallic acid) AND (neurodegeneration or neurodegenerative or neuroprotection or neuroprotective or Alzheimer or Parkinson). On the other hand, auxiliar searches were performed in order to elaborate further on several issues such as specific neurodegenerative hallmarks and certain plants.

### 2.2. Inclusion and Exclusion Criteria

A total of 2599 publications resulted from the core search. Only articles from 2018 to 2022 written in English language were firstly identified. Then, the result was refined using specific citation topics and research categories (phytochemicals, neurodegenerative diseases, neuroscience and food science and tech., cell biology, chemistry, biochemistry, nutrition and dietetics and plant sciences). In terms of the Open Access Status, papers categorized as “Bronze” were rejected since the licensing for these articles was either unclear or nonexistent. Additionally, “Green Submitted/Accepted” statuses were rejected, since the then readable version was not definitive. After this, the title and abstract screening took place, taking into account the citation score. Different issues were considered for rejection: isolated compounds, extracts from other species or by-products (Fungi kingdom, juices and infusions, etc.), other biomolecules (peptides, fatty acids, sugars), focus on other close pathologies (obesity, diabetes) and too generalist or redundant information. Resulting articles were revised, and those that did not specifically cover neuroprotection or that did not provide particularly relevant information due to the nature of the study were excluded.

70 articles were obtained and used for the investigation. Among selected articles there were in vitro and in vivo studies, so they were gathered into different tables for a better understanding. In addition, in silico and ex vivo studies were also considered. Several articles which were included did not fulfil the established criteria according to publication date; regardless, they were included for their relevance to the study.

For in vivo articles, a PICO framework (Patient or Population, Intervention, Comparison and Outcome) was established. Humans and all types of animals of any gender and age were considered valid for the review. In terms of diseases, patients who suffered from AD or PD, or from any pathology or symptom related to neurodegeneration, such as induced oxidative stress or neuroinflammation, were preferred. On the other hand, drug treatment or screening tests using any type of control (placebo, no treatment, standards, etc.) were chosen as the most interesting interventions for this work. Finally, as expected outcomes, we were looking for a downregulation of neurodegeneration through any mechanism involved in one of the different AD and PD hallmarks: oxidative stress, protein aggregation, mitochondrial dysfunction, apoptosis, neuroinflammation and excitotoxicity.

## 3. Pathogenesis and Main Biological Mechanisms of Neurodegeneration

Neurodegenerative diseases can be defined as a heterogenous group of disorders caused by progressive, long-lasting degradation and loss of neuronal cells in specific areas of the central nervous system (CNS) [13]. This leads to the chronic generation of deficits in specific brain functions, such as memory, movement and cognition, finally promoting dementia and neuronal death. Among neurodegenerative diseases, AD and PD are the most common, followed by Huntington’s disease and amyotrophic sclerosis [6]. Due to the complexity of this field, only AD and PD were taken into account for this research.

Dementia is considered to be the loss of mental abilities that affects daily life. This pathology is usually promoted by other neurodegenerative diseases, such as AD (60–80% of all cases). AD is commonly characterized by an unavoidable loss of neurons, formation of neurofibrillary tangles, tau protein aggregation, amyloid β-protein (Aβ) deposition and low levels of acetylcholine (ACh) [10]. Its common symptoms are memory loss, inability to learn new things, loss of language function, impaired perception of space, inability to perform calculations, depression, delusions, etc. [24]. This disease is considered the fifth leading cause of death among the elderly population [2]. PD affects 1–3% of the total population and is characterized by slow and progressive degeneration of dopaminergic neurons in the substantia nigra, overactivation of microglia cells and subsequent neuroinflammation, grey matter cell death in brain and cerebral cortex degeneration [12]. This translates into motor dysfunction, mood alterations and cognitive impairments.

Molecular mechanisms in neuropathologies still represent a complex field of research due to limited knowledge, low financial resources and difficulties in accessing the human brain [8]. However, through in vivo and animal models it has been possible to decipher different questions. There is a common belief that CNS degradation is closely related to abnormal misfolding, protein deposition and, eventually, neuronal death [25]. However, protein aggregation triggers other processes that also contribute to neuronal, synaptic and cognitive deterioration, always regulated by genetic, environmental and endogenous factors related to aging [26]. Main key biological mechanisms observed are neuroinflammation, oxidative stress and increased free radical formation, excitotoxicity, impaired bioenergetics, mitochondrial dysfunction and unbalanced cell apoptosis [17,25]. Thus, neurodegeneration can be considered a multifactorial cyclical problem.

**Figure 2:**
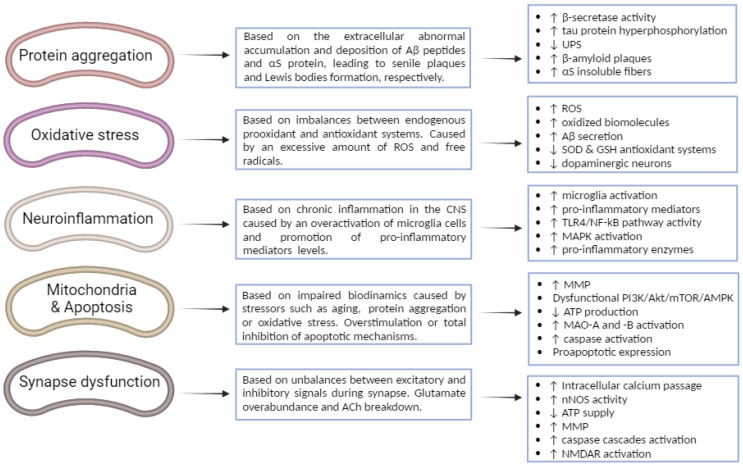
Different key biological mechanisms associated with the neurodegeneration implicated in the progression and pathogenesis of AD and PD, as well as the main cellular processes involved. ↑: increase; ↓: decrease.

Hereunder, the main aspects of each biological mechanism are gathered, accompanied by a summary diagram where key cellular processes are represented (Figure 2).

### 3.1. Protein Misfolding, Aggregation and Deposition

Protein abnormal interactions such as misfolding, aggregation and deposition are considered common pathological hallmarks of neurodegenerative disorders.

Amyloid-β (Aβ) peptides perform different functions in the CNS, and is progressively accumulated in the extracellular space of the brain as a consequence of the sequential cleavage of amyloid precursor proteins (APP) by β-secretase activity [27]. The overproduction of insoluble Aβ, common in AD, forms toxic oligomers which eventually accumulate in senile plaques, which are deposited throughout the brain [2,28]. This condition is usually accompanied by damage to the tau proteins, peptides responsible for the structural support of microtubules in neurons. In this sense, Aβ plaques cause a hyperphosphorylation of tau proteins, which translates into the formation of new neurotoxic tau tangles, known as neurofibrillary tangles (NFT) [27]. This event causes synaptic impairment, induced inflammation through microglia activation and, ultimately, cell death [9,29]. Moreover, the ubiquitin–proteasome system (UPS), the main intracellular proteolytic system which regulates the production and degradation of proteins, is also affected. Its dysfunction, caused by senile plaques and NFTs, eventually leads to further aggregation [26]. Ubiquitinated proteins have previously been detected in senile plaques and NFTs, demonstrating the interrelationship between UPS dysfunction, protein aggregation and deposition inside and outside the neuron [28].

Amyloid plaques are capable of passing to the brain through the blood, damaging neurons and activating different nervous cells such as microglia cells and astrocytes. These events are closely related to other phenomena such as high free radical production, excitotoxicity, increase in neuronal apoptosis and promotion of the synthesis of proinflammatory molecules, which leads to neuroinflammation [2,30,31].

Notwithstanding, new studies are proving that, in contrast to the extracellular deposition of Aβ as AD main initiator, these plaques are indeed the result of intraneuronal accumulation of Aβ inside brain cell lysosomes [32]. Due to the novelty of the finding, the information is scarce, but sets a new point of view for the study of AD neurodegeneration and its mechanisms.

In PD, dementia can be promoted by the spread of alpha-synuclein (αS), a protein that normally originates in the enteric nervous system, with subsequent spread to the rest of the CNS [33]. Physiological functions of this peptide include synaptic vesicle recycling, neurotransmission and synaptic plasticity, among others. Although it is usually soluble, αS can accumulate and form insoluble fibrils that, associated with molecules such as ubiquitin, form clusters known as Lewis bodies [33]. These formations can cause synaptic impairments, mitochondrial dysfunctions, membrane disturbances and neuroinflammation through microglia activation. The spread of αS throughout the system allows it to interact with biological and pathological proteins such as Aβ and tau, creating a vicious cycle that enhances neurodegeneration [34].

### 3.2. Oxidative Stress

Oxidative stress is a deleterious condition caused by imbalances between endogenous pro- and antioxidant species. It is one of the most multifaceted disease triggers in the human body and one of the most important biological mechanisms associated with neurodegeneration in AD and PD [35,36].

Reactive oxygen species (ROS) are chemically reactive molecules produced as by-products derived from the mitochondrial aerobic respiratory chain [23]. In balanced concentrations, they exert essential physiological functions, especially as messenger intermediates in cell signaling processes such as inflammation, cell survival, immune response and synaptic plasticity [37,38]. Nevertheless, an overproduction of ROS can overcome the endogenous antioxidant defense system, composed mainly of the superoxide dismutase (SOD) and the glutathione (GSH) systems. This leads to the deleterious condition of oxidative stress, which affects cell functions and damages different biomolecules through protein oxidation, DNA degradation and lipid peroxidation (LPO) [13,38]. The CNS is particularly vulnerable to oxidative stress due to the high levels of oxygen consumption it requires for a proper functioning, the weakness of its antioxidant system, a limited cellular regeneration capacity and a high content of polyunsaturated fatty acids in neuronal membranes, which are prone to oxidation [36,37,38].

There are different sources of ROS overproduction. Mitochondrion malfunctioning during ATP molecule supply, normally caused by aging or other stressors, is thought to be the main reason. This unbalances the redox system and causes oxidative damage throughout the system [36]. In turn, mitochondrial DNA is also quite sensitive to oxidation, which provokes an even bigger ROS production and leads to a new cyclic problem [39].

Inflammatory responses triggered by brain protein aggregation are considered another important intracellular ROS source in the CNS [39]. In AD, ROS presence oxidizes biomolecules such as proteins, DNA and carbohydrates, which are gradually accumulated in the cell over the years. Neurons, in an attempt to prolong cell life, promote Aβ secretion to sequester ROS, causing their oligomerization and aggregation [40]. Aβ, in turn, is directly involved in ROS formation through peptidyl radicals, metal association or indirect activation of microglia [37]. Therefore, high levels of ROS can be related to neurodegeneration, but they are not considered to be a direct initiator of neuropathies [41,42,43].

In PD, the loss of dopaminergic neurons is closely related to the presence of large amounts of ROS and free radicals through neuroinflammation, dopamine degradation, mitochondrial dysfunction, aging and GSH depletion, among others [37]. Dopamine synthesis is closely related to intracellular oxidation, which makes dopaminergic neurons in substantia nigra especially sensitive to oxidative stress [44].

### 3.3. Neuroinflammation

Neuroinflammation is a pathological process characterized by chronic inflammatory reactions in the CNS which highly contributes to the development of a neurodegenerative state in the brain, mainly AD and PD [26].

Inflammation is a biological mechanism used by an organism against the appearance of an injury or infection through the physiological production of pro-inflammatory markers. However, if this impairment is not corrected in a timely manner, several chronic conditions may be promoted, causing negative health effects [45]. The immune system plays an essential role in this. Microglia represent the most present innate immune cells in the brain and influence other nervous cells such as astrocytes and neurons. Under normal conditions, microglia are deactivated and exert anti-inflammatory responses, but when invaded by pathogens or tissue injury, they are activated. Here, pro-inflammatory responses are promoted, initiating tissue repair cascades that are self-controlled once the repairment has been performed. However, persistent stimulus of altered genes or endogenous factors, such as protein aggregates or ROS overproduction, can confuse the immune system causing uncontrolled inflammation, which generates neurotoxic mediators such as cytokines and interleukins (ILs) that enhance neurodegeneration [44,46].

In AD, the inflammatory response is primarily initiated by senile plaques and NFTs, which trigger inflammatory activation of the closest glial cells through different cascades. The main signaling pathways are the toll-like receptor-4 (TLR-4) pathway, transmembrane proteins expressed in neurons and glial cells involved in innate immune responses via activation of microglia and regulation of pro-inflammatory molecule release, and the mitogen-activated protein kinase (MAPK) pathway [47,48]. Once those microglia and astrocytes near the senile plaques are activated, the release of inflammatory mediators occurs, promoting the secretion of cytokines, chemokines, nuclear factor-Kb proteins, tumor necrosis factor-α and -β, enzymes that metabolize arachidonic acid, such as 5-lipoxygenase (5-LOX) and cyclooxygenase 2 (COX-2), and other compounds such as ROS and glutamate, whose excessive formation induces chronic neuroinflammation and cell death [49]. COX-2 upregulates glial cell activity and synthetizes important pro-inflammatory mediators such as prostaglandins, and LOX-5 catalyzes the formation of leukotrienes, lipid mediators of inflammation. The presence of high levels of lipoxygenase has been related to Aβ overgeneration and tau hyperphosphorylation [9,50].

In PD, the formation of Lewis bodies also promotes the activation of non-neuronal cells, microglia and astrocytes. In addition, αS itself has direct proinflammatory activity. Oligomeric depositions of this peptide are phagocytosed by microglia, followed by their activation, and ROS and NO production, mostly produced by NADPH oxidase in activated microglia [44,51]. Proinflammatory cascades then take place in the same way as explained above.

### 3.4. Impaired Biodynamics and Mitochondrial Dysfunctions

Mitochondria are one of the most important organelles in cells. Their essential role in body homeostasis is related to their capacity to produce energy through ATP formation and regulate calcium homeostasis, their redox signaling and cellular senescence [38]. Due to the high energy demands of the brain, the functions of the mitochondria are especially crucial in neurons for proper synaptic connections and Ca^2+^ storage [52]. Since it is the only organelle with its own genome (mtDNA), mitochondrion is quite sensitive to stressors such as aging and protein aggregation, which promotes mtDNA mutation and a decrease in ATP production [53]. Dysfunctional energy production also affects to the ionic gradients across membranes, which ultimately leads to mitochondrial membrane depolarization and permeabilization (MMP), causing devastating neurological effects [38,39].

Mitochondria control programmed cell death, as mitochondrion can induce apoptosis via caspase mechanisms through different proteins [53]. Apoptosis is normally triggered intrinsically by the mitochondria. Once the apoptotic signal pathway is activated, mitochondrial membrane permeability increases and results in the formation of the mitochondrial permeability transition pore. It allows the release into the cytoplasm of pro-apoptotic cytochrome c or caspase activators. Finally, after translocation to the nucleus, the DNA is fragmented, leading to cell death [9,54]. Mitophagy is the apoptotic mechanism of mitochondria to remove damaged areas through self-phagocytosis, and is regulated by the PI3K/Akt/mTOR/AMPK signaling pathway [55]. The process is promoted by AMPK activating ULK-1; however, mTOR can phosphorylate ULK proteins and prevent autophagy. In addition, Akt is able to regulate apoptosis by directly influencing the release of cytochromes and the consequent activation of caspases. AMPK also works as a cellular energy sensor as it is activated by a decrease in ATP; then, autophagy is enhanced by AMPK upregulation or mTOR downregulation [55,56].

Dysfunctional and unbalanced apoptosis sets a starting point for the development of neurodegenerative diseases [53,57]. In AD, Aβ plaques tend to accumulate and aggregate in the mitochondria, interacting with them and blocking electron transport, compromising ATP production and synapsis, in addition to causing cytochrome C release, all proapoptotic signals [38]. In PD, the accumulation of αS oligomers causes MMP, closely related to the enhancement of ROS and the hyperactivation of glial cells. As a result, the neuronal nitric oxide synthase (nNOS), responsible for the production of NO messengers, is wildly activated, worsening pathological conditions. Mitochondrial impairments also affect skeletal muscles and platelets cells, which explains the progressive motor impairment suffered during PD [53]. All of the above has been considered the primary mechanism of dopaminergic neurodegeneration in PD [58].

On the other hand, bound to the outer membranes of mitochondria, there are monoamine oxidase enzymes (MAO), responsible for neurotransmitter inactivation. In fact, MAO-A and MAO-B can degrade dopamine, which exerts vital roles in the regulation of movement. Therefore, their overactivation can lead to dangerous levels of dopamine depletion, promoting mobility impairments and PD [59].

### 3.5. Excitotoxicity—Glutamatergic and Cholinergic Neurotransmissions

The balance between excitatory and inhibitory neuronal synapsis is important for the healthy state of the CNS. These excitatory signals are mainly regulated by glutamate, the predominant neurotransmitter [60]. Glutamatergic neurons are involved in the proper functioning of several cognitive, motor, sensory and autonomic activities, so their levels must be maintained at a physiological level to avoid neuronal impairments. Acetylcholine (ACh) is another important neurotransmitter involved in nerve–impulse transmission between cholinergic neurons. It is also involved in a large number of physiological processes, such as attention, learning, memory, stress response, wakefulness and sleep and sensory information [61]. Therefore, an overabundance of excitatory neurotransmitter levels within the synapse, known as excitotoxicity, has damaging effects on the CNS, as does a depletion of them [62,63].

In average neuronal activity, glutamate is released from its vesicles into the synaptic cleft. Then, it diffuses across and binds to ionotropic glutamate receptors, such as the N-methyl D-aspartate receptor (NMDAR), responsible for forming ion channels that cause cation influx and enable basis neuronal communication [64]. An altered glutamate homeostasis can be achieved through different mechanisms. In AD, senile plaques and/or oxidative stress are able to disturb glutamatergic networks and lead to glutamate overproduction and NMDAR overactivation [65]. This provokes malfunctions such as the excessive intracellular calcium passage, the enhancement of nNOS activity and the promotion of mitochondrial damage with inhibition of the respiratory chain, ATP depletion and necrotic cell death. Furthermore, intramitochondrial calcium is generated, enhancing MMP and leading to caspase cascade activation and apoptosis [66].

In PD, alterations in glutamatergic neurotransmission have been shown to be important triggers of the disease, since glutamate intervenes in several fronto-basal circuits involved in the modulation of voluntary movements [60]. Consequently, dopaminergic neurons begin to degenerate, leading to excessive promotion of glutamatergic concentration and, as abovementioned, to overactivation of NMDAR. These excitotoxic effects are accompanied by increased ROS levels, which increase neuroinflammation and exacerbate damage to nigral dopaminergic neurons in a vicious cyclical problem [66]. Nowadays, it cannot be determined whether inflammation is a cause or a consequence of glutamate unbalance [60].

On the other hand, the loss of cholinergic neurons is also a common hallmark in neurodegenerative diseases such as AD [67]. A consistent ACh depletion and decline in choline acetyltransferase activity are considered to play a key role in learning and memory deterioration in AD patients [68]. This depletion is caused by overactivation of acetylcholinesterase (AChE), a key enzyme in the cholinergic synapsis. Under normal conditions, AChE is responsible for ACh regulation, turning it into choline and acetate molecules. However, overexposure to stressors such as ROS may motivate AChE activity, deplete ACh concentrations to neurodegenerative levels and promote neurological disorders such as dementia [69]. In addition, the strong connection established between AChE and Aβ proteins has been demonstrated; AChE binds to amyloid proteins through its peripheral anionic site, which promotes protein misfolding, fibril formation and neurotoxicity enhancement [50].

## 4. Phenolic Compounds from Fruit and Vegetable By-Products: Neuroprotective Mechanisms for Potential Biological Targets

Despite advances in addressing the main mechanisms of neurodegeneration, there is still a serious lack of resources and treatments, and current therapies take little account of the multifactorial behavior of neuronal disorders, causing low efficacy and multiple side effects [70]. Modern medicine works on developing new natural therapeutic agents due to their extremely vast array of biological activities and health benefits [71]. In this sense, phenolic compounds from plants have been demonstrated to possess great bioactivity, either as free radical scavengers, hydrogen atom donors or metal ion chelators [23].

Regarding neuroprotection, phenolic compounds show outstanding bioactivity in numerous pathogenic processes related to aging and neurodegeneration: downregulation of oxidative stress and pro-inflammatory cytokine expression, apoptosis regulation, and activation of proteolysis pathways such as the UPS for preventing protein aggregation, among others [9,12,54]. Flavonoids have been proved to prevent ROS formation and promote antioxidant protein expression, neuron viability and cerebral blood flow, reduce apoptosis, amyloidogenic effects and loss of dopaminergic neurons [72]. Phenolic acids have been demonstrated to ameliorate epilepsy, neuroinflammation, apoptosis, memory impairments, excitotoxicity and depression, among others [73].

These compounds, apart from their biological activity, stand out for their ubiquity in plants. Their presence in all kinds of fruits, vegetables and plant material has been extensively reported [74,75]. Over the last few years, by-products such as seeds, peels and leaves are gaining attention for their content of bioactive compounds, which is sometimes higher than in the edible parts [76]. However, as mentioned before, these by-products are usually discarded, creating nearly 30% of food waste annually [23].

According to the abovementioned evidence, the administration of phenolic compounds highlights a potential alternative for the treatment of neurodegenerative pathologies. Therefore, the consumption of fruits and vegetables could be therapeutic due to the presence of these phenolic compounds. Their isolation could be a solution, but the direct application of plant extracts could reduce effort, contamination and costs maintaining the same neuroprotective effect, or even more since some extracts exert their effect thanks to the synergistic effect of all their bioactive compounds [77]. Moreover, it would be a great opportunity for the proper revalorization of agri-food by-products, promoting circular bioeconomy of fruit and vegetables.

All pathophysiological mechanisms abovementioned have been studied not only as neurodegenerative disease drivers, but also as feasible therapeutic targets for phenolic compounds and plant extracts rich in phenolics and other bioactive compounds, to exert their neuroprotective activity. In this regard, studies related to this topic are summarized in Table 1 and Table 2, for the in vitro and in vivo evaluation of neuroprotection, respectively. These tables include information about the type of by-product, the main phenolic families found in their matrices, the biological target and the mechanism through which the therapeutic effect is conducted. In addition, the final neuroprotective effect is remarked. Then, main mechanisms through which phenolic compounds exert the neuroprotective effect are shown, in order to relate them to the composition of plant extracts and conclude the reason why these extracts are interesting and feasible neuroprotectors.

### 4.1. Neuroprotection against Abnormal Protein Deposition

**Figure 3:**
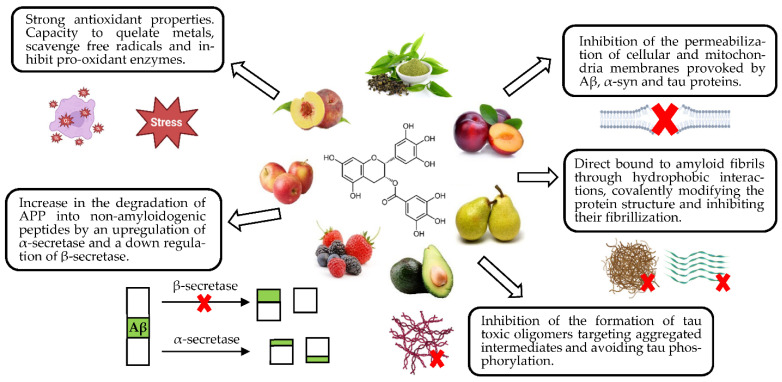
Neuroprotective molecular targets of EGCG in amyloidogenic pathologies.

Phenolic compounds have been shown to prevent protein deposition through numerous mechanisms, using one or more at a time. For example, EGCG has been theorized to protect against protein misfolding through antioxidant properties, degrading APP by α-secretase into non-amyloidogenic proteins, direct binding to the protein avoiding its fibrillization, or even preventing tau aggregation and hyperphosphorylation [9,132,133]. Different mechanisms reported for this compound are gathered in Figure 3.

Polyphenols are known to reduce β-secretase activity and subsequent production of insoluble proteins, such as oleuropein, quercetin, curcumin and EGCG [9]. Through APP cleavage by α-secretase, these compounds cause the inhibition of the amyloidogenic pathway and protect the CNS from the accumulation of Aβ plaques [9,132,133,134,135].

Furthermore, phenolic compounds are able to interact with Aβ and αS hydrophobic sequences breaking β-sheet motifs, preventing tau hyperphosphorylation and making proteins smaller and nontoxic [9]. Quercetin, resveratrol, oleuropein, luteolin, myricetin and ECGC, among others, are able to form hydrogen bonds with β-sheet structure, inhibiting the aggregation of Aβ and preventing tau primary conformation self-assembly [132,133,136]. Structural conformation (≥2 phenolic rings and ≥3 OH groups) is considered a more limiting factor to amyloid and synuclein accumulation than oxidative conditions for an optimal 3D conformation for non-covalent bonds with β-sheet structures [101,137].

Other phenolics promote protein solubility modifying their hydrophobicity [138]. Das et al. (2016) reported for the first time that punicalagin (ellagitannin) interacts directly with Aβ motifs to exert neuroprotective effects [136]. Knowing that punicalagin is the major polyphenolic compound in pomegranate extract, the anti-amyloidogenic effects observed by Essa et al. (2015) and Morzelle et al. (2016) of pomegranate peel and pulp extracts could be explained by punicalagin presence [30,123]. The same applies to olive waste such as leaves, whose oleuropein content is enough to potentially be considered interesting extracts with neuroprotective effects, as reported by Chiaino et al. (2020) [41].

Concerning tau hyperphosphorylation, phenolics such as ECGC and myricetin have been shown to block the phenomenon inhibiting the different phases of fibril assembly, e.g., myricetin is able to interfere in the elongation phase [138]. Quercetin can also perform this anti-hyperphosphorylation effect, which is consistent with Infante-García et al.’s (2017) paper. Here, mango leaf extract, reported to possess quercetin and high amounts of polyphenols in its matrix, inhibited tau hyperphosphorylation [102].

Antifibrillization properties from phenolics maintain a close structure–activity relationship [139]. Ortho-dihydroxyl in flat, planar molecules with substituted aromatic end groups covalently binds to αS side chains, remarkably decreasing its hydrophobicity and detoxifying the protein assembly [132,139]. For example, Gu et al. (2017) were able to demonstrate that mulberry extract reduced α-synuclein levels in Lewis bodies in mice with PD [108]. This effect is probably due to the presence of quercetin in the fruit matrix and its capacity to inhibit αS fibrillization [132]. The same applies to Mohammad-Beigi et al. (2019), who reported olive extract antifibrillization activity and highlighted the extract as a plausible neuroprotective strategy [34].

Finally, Gu et al. (2017) also reported the ability of mulberry extract to upregulate ubiquitin levels, probably due to the high presence of polyphenolic compounds (rutin, quercetin) and different phenolic acids (chlorogenic, caffeoylquinic acids), but with no clear mechanism [108,109]. This probably promotes UPS activity and the normal regulation of proteolysis.

**Figure 4:**
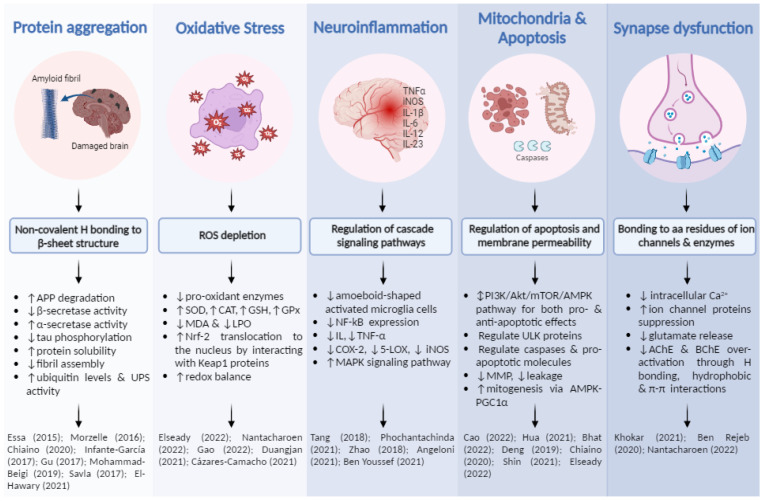
Main mechanisms through which plant extracts and their phenolic compounds exert their neuroprotective effects, as well as key cell processes involved. Finally, several references in which this information is demonstrated [30,34,41,48,57,58,75,79,80,81,85,86,87,88,89,92,93,95,102,108,110,115,123,130,131].

Other studies where the neuroprotective activity from fruit and vegetable by-product extracts are mentioned now. Pasinetti et al. (2010) exposed the role of grape seeds (rich in proanthocyanidins) in blocking Aβ fibril formations by preventing protofibril formation, pre-protofibril oligomerization and initial coiling through β-sheet structure binding, as well as a reduction in tau peptide aggregation [24]. El-Hawary et al. (2021) detailed how different acids from *Morus macroura* pulp and leaf extract (chrysin, resveratrol and ferulic acid) were able to inhibit abnormal Aβ aggregations, blocking β-secretase activity through conformational features and hydrogen bindings [85]. Additionally, they demonstrated that resveratrol and ferulic acid were able to pass through the BBB and reach their target easily. Main mechanisms are shown in Figure 4.

### 4.2. Neuroprotection against Oxidative Stress

Polyphenols from plants are great antioxidants, mainly due to their capacity to modulate multiple cellular processes, such as redox balance. Knowing how antioxidant systems are affected in neuropathological conditions, the use of plant extract rich in phenolics could ameliorate neurodegenerative processes [140]. The antioxidant potential of phenolic compounds is conducted through three different mechanisms: suppressing enzymes or chelating metal ions which promote ROS, scavenging ROS and upregulating endogenous antioxidative systems [53,55]. Usually, hydroxyl group number and position on the aromatic ring are quite important conditions, which allow flavonoids to be singularly effective antioxidants [55,141]. On the other hand, carotenoids have also been proved to improve the activity of the endogenous antioxidant system, an effect observed by Elseady et al. (2022) in saffron stigma carotenoid-rich extract [53,130].

Polyphenols such as resveratrol, curcumin and ECGC, among others, have been reported to protect against neurodegeneration through the activation of signaling molecular pathways such as Keap1/Nrf-2/ARE, which is the main protective pathway against endogenous and exogenous ROS [140,142]. Here, the Keap1-Nrf-2 complex interacts with the phenolics through the active site of Keap1, provoking its disaggregation and subsequent translocation of Nrf2 to the nucleus, where it triggers the expression of antioxidant molecules such as GSH and HO-1 [140,143]. In addition, another effective mechanism is the inhibition of molecules that are especially sensitive to the oxidative stress state, such as nuclear factor-kB (Nf-kB). Gao et al. (2022) reported an increase in GSH, SOD and CAT activities and a decrease in MDA production after a pre-treatment with *Vaccinium dunalianum* fruit, leaf and flower extracts, which contained excellent amounts of polyphenols such as kaempferol and feruloylquinic acid [95].

Flavonoids have also been demonstrated to prevent ROS formation by modulating the cell signaling pathway Keap1/Nrf-2/ARE [72]. Apart from that, they are able to reduce amyloidogenic effects, dopaminergic neuron loss, apoptosis, etc. This seems to be related to their ability to form ligands with receptors in the CNS, in addition to the fact that their structure offers them a lipophilic character which allows them to cross the BBB [72]. Nantacharoen et al. (2022) also reported that *Cleistocalyx nervosum* fruit extract showed antioxidant activity upregulating the gene expression of different cellular antioxidant enzymes, such as SOD, CAT and GPX, promoting the translocation of the Nrf2 protein from the cytoplasm into the nucleus. Additionally, resveratrol found in their extract could be one of the main responsible factors of this effect, as previously reported [86].

Therefore, flavonoid-enriched extracts could show significantly high antioxidant levels, as several studies confirm, either by promoting endogenous antioxidants or scavenging free radicals [75,83,87]. For example, Duangjan et al. (2021) reported that grape leaf extract promoted the gene expression of different cellular antioxidant enzymes such as CAT, SOD, GST and GPX in vivo [87]. Resveratrol, quercetin, apigenin and catechin can be highlighted as the most common reported phenolics in grape leaves, which could be provoking this antioxidant effect. The presence of resveratrol could be the main mechanism through which endogenous antioxidant enzymes levels are promoted, due to the promotion of Nrf2 expression and translocation. Indeed, it was reported that the extract’s ability to promote the translocation of DAF-16 in *C. elegans*, had the same result as Nrf2 translocation.

Other studies demonstrated the ability to downregulate lipid peroxidation, one of the most damaging symptoms of oxidative stress, by reducing levels of MDA [91,123]. All reported biological processes examined are gathered in Figure 4.

### 4.3. Neuroprotection against Neuroinflammation

Polyphenols have been shown to exhibit numerous anti-neuroinflammatory activities. One such example is the downregulation of microglial activation and subsequent release of proinflammatory factors, such as TNF-α, IL-6 and iNOS, through the inhibition of cellular cascade signaling pathways involving NF-kB via nuclear translocation of certain subunits [144,145]. Moreover, there is the inhibition of the activity of COX-2 and LOX-5 enzymes. In third place, there is the attenuation of phosphorylation and overactivation of mitogen-activated protein kinases (MAPKs), such as IkB and p38 MAPK, to protect against dopaminergic neuronal death [144]. Finally, there is the avoidance of TL receptor overexpression, such as TLR4 [145].

The mechanisms through which phenolic compounds prevent chronic neuroinflammation are still scarcely studied. However, the modulation of the NF-kB signaling pathway seems to be one of the most important. NF-kB is a transcription factor that regulates the expression of almost 500 different genes, including pro-inflammatory enzymes and cytokines [146]. Thus, the constant activation of NF-kB in chronic inflammation leads to detrimental effects. Ben Youssef et al. (2021) reported that grape seed and skin extract, especially rich in resveratrol, catechins and gallic acid, inhibited the NF-kB pathway, probably reducing the activity of its subunits [115].

Polyphenols are also theorized to modify morphological features of microglial cells. Tang et al. (2018) found that fully activated microglial cells showed an amoeboid shape. Catechin and procyanidin A2 obtained from lychee seed extracts (LSE) reduced microglia activation by decreasing amoeboid-shaped microglia proportions in cell cultures [80]. These polyphenols were also found to downregulate NF-kB expression and apoptosis. Numerous studies reported this ability to reduce microglia burden and overactivation, reducing the proportion of amoeboid-shaped glial cells [102,120,122].

Zhao et al. (2018) also showed that LSE reduced mRNA levels and the protein expression of pro-inflammatory mediators (IL-1, TNF-α, COX-2, iNOS). Furthermore, the extract was able to attenuate IkB phosphorylation and keep NF-kB inactive [79]. LSE was rich in procyanidins and was probably responsible for the great neuroprotective effect exerted. Phochantachinda et al. (2021) studied the anti-neuroinflammatory potential of Indian gooseberry pulp, which appeared to reduce the release of proinflammatory cytokines IL-6 and TNF-α after inducing microglia activation. Additionally, it promoted neuronal differentiation by inducing the expression of neuronal markers from the MAPK signaling pathway, which modulated the growth and stabilization of microtubules in neurites [48]. Angeloni et al. (2021) reported that coffee by-product extract was able to reduce the activity of pro-inflammatory enzymes, such as iNOS and COX-2, and the levels of different pro-inflammatory mediators by reducing NF-kB proteins to basal levels [89]. In fact, these coffee extracts showed catechin content, in accordance with LSE. A high number of reports have demonstrated brain anti-inflammation through the downregulation of pro-inflammatory cytokines IL-6 and TNF-α, among others, and enzymes such as COX and LOX [42,84,124,129,130]. Key cellular processes are shown in Figure 4.

### 4.4. Neuroprotection against Mitochondrial Dysfunctions

Polyphenols are capable of preventing or promoting apoptosis according to necessity, enhancing mitochondrial biogenesis, influencing mitochondrial fission and fusion, affecting mitophagy, controlling mitochondrial quality and regulating mitochondrial functions such as ETC and ATP synthesis [53,54]. The PI3K/Akt/mTOR/AMPK pathway is an interesting biological target for polyphenols to act as both pro- and anti-apoptotic mediators [10,128,147]. Resveratrol has been reported to achieve inhibition of the mTOR complex that competes against ATP for the ATP-binding sites of mTOR [148]. Oleuropein from olives has been also found to trigger autophagy, activating AMPK [149]. Curcumin has been proved to inhibit the Akt/mTOR pathway to induce protective autophagy [147].

Cao et al. (2022) reported that a passion fruit pericarp extract exerted neuroprotective effects through stimulation of mitophagy by inhibiting mTOR and directly activating ULK1 proteins [57]. Passion fruit characterization in previous reports has shown resveratrol and curcumin in its matrix, which could be the reason for this neuroprotective effect [150]. On the other hand, after intoxication or ischemia, neuronal death is harshly triggered throughout the CNS, causing irreparable damage [92]. Therefore, anti-apoptotic activity is needed. Hua et al. (2021) stated that wild turnip root extracts exerted anti-autophagic activity by restoring phosphorylated PI3K, Akt and mTOR levels, which can be translated into an activation of the PI3K/Akt/mTOR pathway and a promotion of cell survival [92]. Wild turnip has been previously reported to possess different phenolic acids in its matrix, such as ferulic and caffeic acids, compounds which have been proven to modulate different signaling cascades related to cell survival and proliferation [151,152]. Bhat et al. (2022) also showed the ability of velvet bean seed extract to promote Akt activity through the PI3K/Akt/mTOR pathway, preventing cell apoptosis and repairing induced damage [97]. Moreover, this extract regulated neurotransmitter levels by reducing MAO activity.

Mitochondria biogenesis (mitogenesis) is a metabolic process dedicated to maintaining the organelle’s integrity. Its sensitive relation to oxidative stress turns mitochondria dysfunction into one of the main pathogeneses of neurodegeneration. Polyphenols are able to upregulate the AMPK-PGC1α signaling pathway responsible for mitogenesis: Deng et al. (2019) previously reported on the behavior of the mitochondrial biogenesis promoter shown by ginger extracts, which increased mitochondrial mass, mtDNA copy number and ATP production, as well as the activity of mitochondrial complexes through the regulation of the AMPK-PGC1α signaling pathway [131].

Other studies have focused on different mechanisms. Chiaino et al. (2020) reported increased cell viability by reducing mitochondrial membrane potential loss and cytoplasmatic caspase levels with an extract of olive leaf and hibiscus flower, probably for the presence of oleuropein in the extract [41]. Shin et al. (2021) showed that mulberry fruit extract, rich in phenolic acids and flavonoids, prevented mitochondria membrane from depolarizing, in addition to suppressing pro-apoptotic factors such as cytochrome c [110]. Finally, Elseady et al. (2022) demonstrated the ability of saffron stigma extract to decrease caspase-3 levels, reducing cell apoptosis [130]. All abovementioned is summarized in Figure 4.

### 4.5. Neuroprotection against Impaired Glutamatergic and Cholinergic Neurotransmissions

Excitotoxicity is considered another biological target of polyphenols, which are able to inhibit glutamate-induced Ca^2+^ increases and intracellular accumulation by suppressing ion channel proteins through hydrogen-bonding with their amino acid residues, reducing triggering signaling cascades and, finally, reducing glutamate release from their vesicles [153,154]. However, no reports have been found relating this to plant extract effects.

Furthermore, the glutamate accumulation in neurons leads to the generation of intracellular ROS and oxidative stress, as well as the diminution of intracellular GSH levels [86]. Thus, polyphenols may be useful for reducing excitotoxic effects, as Nantacharoen et al. (2022) reported. Here, the *Cleistocalyx nervosum* extract, rich in resveratrol, was reported to promote the expression of endogenous antioxidant enzymes and suppress the caspase expression in glutamate-induced oxidative damage models [86].

Phenolic compounds also can inhibit enzymatic disorders that end in neurodegeneration, such as the overactivation of AChE and BChE (butyl cholinesterase). In this respect, the inhibition is also explained by morphological features (active sites and oxyanion holes). Polyphenols are able to interact with amino acid residues of enzymes structures through the formation of hydrophobic interactions, π-π and hydrogen bonds thanks to their methoxy and hydroxyl groups [94,155]. This ability is a function of the number and position of their OH groups [94]. Khokar et al. (2021) studied the ability of different phenolic compounds from pomegranate peel extract in silico to attach to AChE and then inhibit its activity. Catechin was reported to be the most promising anti-AD compound for its low binding energy for AChE [88]. Then, other reports of AChE inhibitory activity where the catechin content was high enough could be also explained [67,78,83]. Ben Rejeb et al. (2020) stated that extracts from artichoke by-products (leaves, bracts and stems) inhibited AChE and BChE activity, and they related this to the presence of flavonoids in a positive and significant way [93]. In fact, they highlighted luteolin and its derivatives as the main contributing factor, and reported that flavonoids such as quercetin were able to block the entrance to the active site of AChE and BChE enzymes.

Some studies were conducted ex vivo, recollecting brain tissue after the consumption of the studied extract, such as Phachonpai et al. (2021), who used wampee fruit peel to attenuate memory deficits in rats and showed that cognitive impairment was reduced by its supply [106]. Main mechanisms and biological processes are gathered in Figure 4.

## 5. Conclusions and Future Perspectives

AD and PD are the most common neurodegenerative conditions suffered by humans as they grow older, and represent major unmet challenges for medicine. In the present scenario, plant by-products have been deeply highlighted as natural antioxidant extracts that allegedly are able to display neuroprotective effects thanks to their phenolic compound-rich compositions. Although this effect has been extensively exposed and reported in different samples, the use of plant extracts as neuroprotective agents is still limited. Main limitations are now exposed:

- Digestion, absorption and metabolization of fruit, vegetables and other plants are crucial for recovering phenolic and other bioactive compounds from their matrices. Their bioavailability is considered one of the most limiting factors for plants to exert neuroprotection, since their effects are positively related to the amount consumed [1,156]. Low absorption rates and quick metabolism could limit their efficacy, but abusive consumption could lead polyphenols to show pro-oxidant activity.
- Circulating metabolite structures usually differ from their native dietary compound, so it could be thought that their activity also differs [18]. Additionally, levels of circulating bioavailable metabolites are always below consumed levels, limiting their neuroprotection even more [157].
- Secondary metabolites from phenolic compounds should be able to pass the BBB to reach the brain. Therefore, their effectiveness also depends on their permeability capacity across this barrier [12].
- Phenolic compounds are not the only compounds in plant matrices, so neuroprotection could be exerted by others too, and may be under synergistic or antagonistic interactions.
- Difficulties in carrying out in vivo studies in humans.

These questions, among others, remain unresolved. Therefore, more research should be conducted in order to elucidate the mechanisms and physiological processes through which fruits, vegetables and plants exert their neuroprotection. Several solutions are currently in progress, such as the encapsulation of plant extracts using biodegradable materials, which promotes the enhancement of phenolic bioavailability and BBB permeability, and their therapeutic efficacy [132].

After this review, results obtained establish a starting point for further research that must be carried out in order to confirm plant extracts’ beneficial effects. Here, a detailed description of the neuroprotective nature of different phenolic compounds and extracts has been exposed, demonstrating their value for the development of innovative neuroprotective strategies.

## Figures and Tables

**Table 1:** Different in vitro studies where fruit and vegetable by-product extracts rich in phenolic compounds (and other bioactives) were tested in neurodegenerative models.

Plant Species	By-Product	Main Compounds	Biological Target	Mechanisms of Action	Neuroprotective Effects	Citation
Avocado *(Persea americana)*	Seed + Peel	Caffeic acid, (epi)catechin, rutin, B- type procyanidins.	AChE inhibition.	Inhibition of AChE activity up to 65%.	Anti-AChE.	[78]
	Seed + Leaf	Polyphenols.	AChE and BChE inhibition.	Remarkable enzymatic inhibition of AChE and BChE, avoiding acetylcholine breakdown and increasing communication between nerve cells.	Anti-AChE/-BChE.	[76]
Passion Fruit *(Passiflora edulis)*	Pulp + Seed	Phenolic acids, flavonoids, stilbenes. Piceatannol.	Downregulation of enzymes.	AChE IC_50_ 29.42 µg/mL and LOX IC_50_ 27.682 µg/mL.	Anti-inflammatory and anti-AChE.	[20]
Lychee *(Litchi chinensis)*	Seed	Saponins.	Apoptosis and MMP.	Suppression of apoptosis in Aβ-induced cells through upregulation of Bcl-2 proteins. Decrease in caspase-3 mRNA expression and nuclear translocation of NF-kB. Improvement in mitochondrial function (MMP decrease).	Anti-apoptotic and anti-MMP.	[31]
		A- and B-type procyanidins, rutin, quercetin, saponins.	Suppression of brain inflammation induced by amyloid aggregation.	Modulation of NF- κB signaling pathway. Reduce of mRNA and protein expressions of IL-1, TNF-α, COX-2 AND iNOS. Inhibition of cell apoptosis in microglial cells overactivated by Aβ deposition.	Anti-inflammatory and anti-apoptotic.	[79]
		Catechin and procyanidin A2.	Suppression of brain inflammation induced by amyloid aggregation.	Inhibition of the neuroinflammatory effect induced by amyloid aggregation and deposition by modulation of NF-kB signaling pathway.	Anti-inflammatory.	[80]
Papaya *(Carica papaya)*	Leaf	Carpaine enriched (alkaloid).	Cytotoxicity and inhibition of Aβ_42_ aggregation.	Stabilization of intracellular microtubules and formation of hydrophobic bind to Aβ_42_ sequence, thus inhibiting Aβ_42_ peptide aggregation.	Anti-amyloidogenic and cell structural stabilizer.	[81]
Red Dragon Fruit *(Hylocereus polyrhizus)*	Pulp + Seed	Phenolic and organic acids, flavonoids and carotenoids.	AChE and BChE inhibition.	Inhibition of 69.11 ± 1.12% against AChE and 64. 78 ± 0.71% against BChE.	Anti-AChE/-BChE.	[82]
Sweet cherry *(Prunus avium)*	Pulp	Organic and phenolic acids, (epi)catechin, quercetin, luteolin and procyanidin B2.	AChE inhibition and antioxidant potential.	Inhibition of AChE activity and high antioxidant capacity.	Anti-AChE and antioxidant.	[83]
Blueberry *(Vaccinium carymbosum)*	Pulp + Leaf	Anthocyanins (Delphinidin and cyanidin).	Neuroinflammatory responses in microglia.	Antioxidant activity, reduction in inflammatory mediator levels.	Antioxidant and anti-inflammatory.	[84]
King white mulberry *(Morus macroura)*	Pulp + Leaf	Phenolic acids, flavonoids, terpenes, sterols, stilbenes and anthocyanins.	Protein accumulation and AChE inhibition.	Inhibition of the abnormal aggregation of Aβ_1-42_ through inhibition of β-secretase (in silico), and downregulation of AChE and MAO.	Anti-secretase and anti-AChE.	[85]
Indian gooseberry *(Emblica officinalis)*	Pulp	Gallic, ellagic, ascorbic and mucic acids, tannins, quercetin and rutin.	Neuroinflammation.	Reduced release of proinflammatory cytokines IL-6 and TNF-α from the microglia activation. Growth of neurite length. Induction of mRNA expression of neuronal markers such as MAP2. Decrease in NO production.	Anti-inflammatory.	[48]
North-Thai Berry *(Cleistocalyx nervosum var. paniala)*	Pulp	Flavonoids and resveratrol	Excitotoxicity. Apoptosis and oxidative stress.	Improvement in cell morphology and survival after glutamate-induced toxicity. Inhibition of apoptosis suppressing cleaved caspase-3 levels. Suppression of intracellular ROS levels and upregulation of mRNA/gene expression of SOD, CAT, GPX1. Stimulation of Nrf2 activity.	Anti-excitotoxic and antioxidant.	[86]
Grape *(Vitis vinifera)*	Leaf	Resveratrol, gallic acid, apigenin, catechin, quercetin.	Antioxidant potential and excitotoxicity.	Low glutamate-induced cell death. Inhibition of ROS accumulation and promotion of CAT, SOD, GST and GPx enzymes.	Antioxidant and anti-excitotoxic.	[87]
Orange *(Citrus sinensis)*	Entire fruit	Mono and sesquiterpenes.	Enzyme inhibition, inflammation and oxidation.	AChE, BChE and LOX inhibition capacity. Significant anti-cholinergic compounds expected to have fewer side effects than synthetics.	Anti-AChE/-BChE and anti-inflammatory.	[42]
Pomegranate *(Punica granatum)*	Peel	Caffeic, chlorogenic, coumaric and gallic acids, catechin, quercetin, delphinidin and cyanidin.	AChE inhibition and in silico studies.	AChE and β-secretase inhibition due to hydrogen bond formation.	Anti-AChE and anti-secretase.	[88]
Pistachio, fig and date	Flower buds	Chrysoeriol, epicatechin, feruloyl-malic, caffeoylquinic and coumarylquinic acids, quercetin, isorhamnetin, etc.	Endogenous antioxidant enzymes in plasma from PD humans.	Significant antioxidant enzymatic properties expressed by the increase in plasma SOD and GR activity. Positive correlations between TPC and GR, and TFC and GR. Demonstration of synergistic effect of phenolics.	Antioxidant.	[77]
Coffee *(Coffea)*	Waste	Gallic, loganic, caffeoylquinic acids, rutin, naringin, quercetin, quercitrin, catechin.	Mitochondrial dysfunction and neuroinflammation.	Increase in cell viability and intensification of mitochondrial respiration. Reduction in iNOS, COX-2 and cytokines expression maintaining NF-kB proteins from TLR4/NF-kB pathway at control levels.	Pro-mitochondrial respiration and anti-inflammatory.	[89]
Pear *(Pyrus communis)*	Pulp + Peel	Arbutin, rutin, chlorogenic acid, quercitrin and procyanidins B1 and B2.	Antioxidant potential and AChE inhibition.	Significant antioxidant activity. Inhibition of AChE.	Antioxidant and anti-AChE.	[90]
Olive *(Olea europaea)*	Entire fruit	Oleuropein.	Protein accumulation.	Inhibition of nucleation, elongation, fibrillation and oligomerization of αSn.	Anti-aggregation.	[34]
Wild plum *(Harpephyllum caffrum)*	Pulp	Nobiletin, puerarin, phellamurin, tetragalloyl glucose, quercetin glucoside, etc.	Antioxidant potential, LPO and AChE inhib. (in silico).	Increase in GSH, SOD, and CAT levels. Reduction in MDA concentration and AChE activity.	Antioxidant and anti-AChE.	[91]
Wild turnip *(Brassica rapa)*	Root	Kaempferol and isorhamnetin, phenolic and organic acids.	Mitochondrial apoptosis.	Avoidance of autophagy promoting phosphorylation of PI3K, Akt and mTOR and activating PI3K/Akt/mTOR pathway on HT-22 cells. Inhibition of ROS and restauration of mitochondrial expression.	Anti-autophagic and antioxidant.	[92]
Artichoke *(Cynara scolymus)*	Leaf, Bract and Stem	Caffeoylquinic, feruloylquinic acids, luteolin derivatives.	Antioxidant potential and AChE inhibition.	Radical scavenging and antioxidant potential. Inhibition of AChE and BChE.	Antioxidant, anti-AChE and anti-BChE.	[93]
Black Pigweed *(Trianthema portulacastrum)*	Leaf	Protocatechuic, caffeic, chlorogenic, and ferulic acids.	Antioxidant potential and AChE inhibition (in silico).	Inhibition of AChE activity, as well as high antioxidant activity. Chlorogenic acid showed most significant binding affinity towards AChE.	Antioxidant and anti-AChE.	[68]
*Senecio biafrae* *(Senecio biafrae)*	Leaf	Gallic, chlorogenic, caffeic acids, kaempferol, rutin, quercetin.	Antioxidant potential and AChE inhibition.	NO radical scavenging, ferric chelating capacity and antioxidant potential. Inhibition of AChE and BChE.	Antioxidant, anti-AChE and anti-BChE.	[94]
Sacred lotus *(Nelumbo nucifera)*	Seed, stalk, leaf, petal and stamen	Gallic, ferulic, p-coumaric acids, naringenin, quercetin, luteolin, kaempferol, cyanidin, etc.	Antioxidant potential, AChE inhibition and protein accumulation.	High TPC and antioxidant values. Ability to inhibit enzymes such as AChE, BChE and BACE-1, avoiding cytotoxic Aβ peptide aggregation.	Antiamyloidogenic, antioxidant and anti-AChE/-BChE.	[69]
Que Zui Tea *(Vaccinium dunalianum)*	Flower, fruit and leaf	Chlorogenic. caffeic, p-coumaroyl and feruloyl quinic acids, kaempferol, eriodyctiol, etc.	Antioxidant potential.	High intracellular ROS scavenging effect. High levels of CAT, GSH and SOD. Downregulation of MDA and apoptosis. Elevated expression of Nrf2.	Antioxidant and antiapoptotic.	[95]
African blackwood *(Dalbergiella welwitschii)*	Leaf	Proanthocyanidins and flavonoids.	Antioxidant potential and AChE inhibition.	High antioxidant activity and radical scavenging capacity. Ability to inhibit AChE and BChE activity.	Antioxidant and anti-AChE/-BChE.	[96]
Velvet bean *(Mucuna pruriens)*	Seed	Isoflavanones and alkaloids.	Antioxidant potential, AChE and neurotransmission.	Good antioxidant activity, reduction in MAO A, B and AChE activities.	Antioxidant and anti-AChE.	[97]
Oat *(Avena sativa)*	Seedling	Feruloylquinic acid, isoorientin, vitexin, avenacosides A and B, diosgenoside, etc.	LPO oxidative stress induction and protein aggregation.	Possible inhibition of LPS-induced ROS in microglia. Significant reduction in Aβ 42 and Aβ 40, reduction in APP levels.	Antioxidant and anti-amyloidogenic.	[98,99]
Sickle Senna *(Cassia tora)*	Leaf	Gentistic, ferulic, p-coumaric, vanillic and gallic acids.	Antioxidant potential, DNA damage and apoptosis.	Prevention of ROS generation, LPO, DNA damage, apoptosis and neuronal cell death.	Antioxidant and antiapoptotic.	[100]
*Elaeagnus glabra* *(Eleagnus glabra)*	Branch	4-hydroxybenzoic, vanillic acids, (epi)(gallo)catechin, procyanidin B type, kaempferol, etc.	Protein aggregation and antioxidant potential.	Strong inhibitory effect on Aβ aggregation. Remarkable radical scavenging activity.	Anti-amyloidogenic and antioxidant.	[101]

AChE: Acetylcholinesterase; XO: Xanthine Oxidase; ·O_2_: Superoxide Radical; ·NO: Nitric Oxide Radical; HOCl: Hypochlorous Acid; LRRK2: Leucin Rich Repeat Kinase 2; ROS: Reactive Oxygen Species; BChE: Butylcholinesterase; LOX: Lipoxygenase; IC_50_: Inhibitory Concentration (50%); MMP: Mitochondrial Membrane Permeabilization; mRNA: mitochondrial RNA; NF-kB: Nuclear Factor Kappa-Light-Chain-Enhancer of Activated B cells; IL: Interleukins; TNF-α: Tumor Necrosis Factor Alpha; COX-2: Cyclooxygenase 2; iNOS: Nitric Oxide Synthase; IkB: IkappaB Kinase; Aβ: β-amyloid; MAO: Monoamine Oxidase; MAP2: Microtubule-Associated Protein 2; αSN: α-synuclein; LPO: Lipid Peroxidation; GSH: Glutathione; SOD: Superoxide Dismutase; CAT: Catalase; MDA: Malondialdehyde; PI3K: Phosphoinositide 3 Kinase; Akt: Protein Kinase B; mTOR: Mammalian Target Of Rapamycin; BACE: β-secretase.

**Table 2:** Different in vivo studies where fruit and vegetable by-product extracts rich in phenolic compounds (and other bioactives) were tested in neurodegenerative models.

Plant Specie	By-Product	Main Compounds	Biological Target	Mechanisms of Action	Neuroprotective Effect	Citation
Avocado *(Persea americana)*	Seed and peel	Caffeic acid, catechin, rutin, procyanidin and flavonoids.	*Drosophila melanogaster* survival and movement.	Increase in the number of flies capable of flying above the limit established.	Anti-AChE.	[78]
	Peel	B-type procyanidins, flavanols monomers and chlorogenic acids.	Antioxidant potential in *Drosophila melanogaster*.	Increase in the life span and locomotor activity, decrease in LPO, prevention of induced locomotor impairment.	Antioxidant.	[22]
Mango *(Mangifera indica)*	Leaf	Iriflophenones, mangiferin, quercetin, galloyls, etc.	Neuroinflammation and protein accumulation in diabetes mice.	Decrease in the inflammatory response and microglia burden, reduction in tau hyperphosphorylation in cortex and hippocampus.	Anti-inflammatory and antifibrillization.	[102]
	Peel and pulp	Gallic, chlorogenic, vanillic acids, mangiferin, etc.	Antioxidant potential and LPO in rats (*ex vivo*).	Increased expression and activity of SOD and GST. Prevention in LPO upregulation.	Antioxidant.	[75]
Passion Fruit *(Passiflora edulis)*	Pericarp	Polyphenols, triterpenoids, glycosides, carotenoids, aromatic oils, etc.	Maintenance of mitochondrial homeostasis in *Caenorhabditis elegans*.	Stimulation of mitophagy through activating protein ULK1, enhancement of the cholinergic neuronal resistance and glutamatergic neurons protection.	Pro-phagocytic and anti-excitotoxic.	[57]
Lychee *(Litchi chinensis)*	Seed	Saponins.	Apoptosis in rats.	Suppression of apoptosis in Aβ-induced cells through upregulation of Bcl-2 proteins and decrease in caspase-3 mRNA expression.	Anti-apoptotic.	[103]
		Rutin, quercetin, catechin and Proanthocyanidins.	BBB dysfunction.	Improvement in spatial learning and memory function. Inhibition of caspase-1 and IL-1 expression, and inhibition inflammasome activation through AMPK/mTOR/ULK.	Pro-autophagocytic and anti-inflammatory.	[104]
Papaya *(Carica papaya)*	Leaf	Flavonols, tannins, alkaloids.	AChE inhibition and antioxidant potential in Wistar rats (ex vivo).	Inhibition of AChE and BChE. High radical scavenging capacity.	Anti-AChE and antioxidant.	[105]
Wampee *(Clausena lansium)*	Peel	Coumarins and others.	AChE inhibition, LPO and antioxidant enzymes evaluation in Wistar rats.	Reduced AChE activity and decreased oxidative status restoring SOD, CAT, and GPx activities. Low LPO.	Anti-AChE and antioxidant.	[106,107]
Sweet cherry *(Prunus avium)*	Pulp	Organic and phenolic acids, (epi) catechin, quercetin, luteolin and procyanidin B2.	Antioxidant potential in *Caenorhabditis elegans*.	High antioxidant in vivo capacity.	Antioxidant.	[83]
Mulberry *(Morus alba)*	Entire fruit	Procatechuic, chlorogenic and caffeolyquinic acids, taxifolin, rutin, quercetin, etc.	Protein accumulation and proteolytic systems dysfunctions in parkinsonian mice.	Protection against induced loss of dopaminergic neurons through reduction in α-synuclein and ubiquitin upregulation levels.	Anti-aggregation and UPS promoter.	[108,109]
		Phenolic acids and flavonoids.	Antioxidant potential. Mitochondria and apoptosis in mice (*ex vivo*).	Improvement in glutathione level. Prevention of mitochondria membrane depolarization. Suppression of pro-apoptotic factors such as cytochrome c. Elevated expression of Akt peptide and Nrf2 translocation.	Antioxidant, antiapoptotic and anti-MMP.	[110]
Mulberry and ginger	Fruit andrhizomes	Flavonoids, gingerol, quercetin, cyanidin, ferulic and gallic acids, etc.	Antioxidant enzymes, AChE inhibition and neuroinflammation on Wistar rats (*ex vivo*).	Cognitive enhancing effect and mitigation of reduction in neuron density. Increased levels of SOD, CAT and GSH, and decreased levels of MDA and AChE activity. Suppression of IL-6 expression.	Antioxidant, anti-AChE and anti-inflammatory.	[111]
Cranberry *(Vaccinium macrocarpon)*	Entire fruit	Anthocyanins, procyanidins A and B, hydroxycinnamic acids and flavonols.	Cognitive health examination of healthy older adults.	Improvement in episodic memory performance and neural functioning maybe through increased regional perfusion in several brain areas.	Memory promoter.	[112]
Grape *(Vitis vinifera)*	Seed	Proanthocyanidins and resveratrol.	Antioxidant potential on albino rats.	Increase in levels of GSH, GPX, SOD and CAT activities. Low MDA levels. Inhibition of LPO. Downregulation of iNOS mRNA expression.	Antioxidant.	[113]
		Proanthocyanidins and resveratrol.	Protein accumulation in a mouse model of AD.	Blockage of Aβ fibril formation interfering with (pre)protofibril formation and oligomerization through β-sheet structure. Reduction in tau aggregations and promotion of α-secretase activity.	Anti-fibrillization, anti-aggregation and anti-secretase.	[24,114]
	Seed and peel	Rutin, gallic acid, (epi)catechins, quercetin, vanillin, resveratrol.	Neuroinflammation in mice.	Protective effect against neurotoxicity. Mitigation of caspases-3 and ROS production. Decrease in NF-kB p65 subunit liberation and translocation. Improvement in motor deficits preventing DA neurons.	Anti-apoptotic, antioxidant and anti-inflammatory.	[115]
	Peel	Gallic, caffeic, ferulic acids, quercetin, rutin, (epi)catechin, ECG, resveratrol, etc.	Protein aggregation, neuroinflammation and antioxidant potential.	Improved spatial learning and memory ability. Reduced levels of BACE-1, β cleavage of APP and inflammatory cytokines. Increased SOD and decreased MDA.	Anti-amyloidogenic, antioxidant and anti-inflammatory.	[116]
	Leaf	Resveratrol, gallic acid, apigenin, catechin, quercetin.	Promotion of endogenous antioxidant systems in *C. elegans*.	Stress resistance and improvement in survival rate. Reduced intracellular ROS levels. Significant increase in DAF-16 nuclear translocation. Promotion of sod-3 and gst-4 gene expression.	Antioxidant.	[87]
Indian gamboge *(Garcinia morella)*	Peel	Morellin, mangostin, cambogic acid, garcinol, etc.	Neuroinflammation and mitochondria dysfunction in parkinsonian albino mice.	Prevention of the loss of nigral dopaminergic neurons. Alleviation of inflammatory marker nNOS and recovery of mitochondrial complex II inhibition.	Anti-inflammatory and mitochondrial alleviator.	[58,117]
Sour orange *(Citrus aurantium)*	Seed	Limonoids and hesperidin.	AChE inhibition and protein aggregation in Wistar rats.	Reduced AChE, tau protein and Aβ levels.	Anti-AChE and anti-aggregation.	[118]
Kawachi bankan *(Citrus kawachiensis*)	Peel	Naringin, narirutin, auraptene, flavones.	Neuroinflammation, antioxidant potential, avoidance of dopaminergic neurons and protein dysfunctions in mice.	Suppression of microglial activation and blocking morphological changes (ameboid shaped) in the activated microglia. Suppression of tau hyperphosphorylation and neuronal death. Increase in total GSH.	Anti-inflammatory, anti-hyperphosphorylation and antioxidant.	[119,120,121,122]
Pomegranate *(Punica granatum)*	Peel	Anthocyanins, ellagic acid, flavanols (catechins and gallocatechins), punicalagin.	Protein accumulation, AChE inhibition and antioxidant potential in mice.	Reduction in amyloid plaque density, increase in neurotrophin BDNF and reduction in AChE activity. Decrease in LPO and in the concentration of pro-inflammatory cytokine TNF-α.	Anti-aggregation antioxidant, anti-AChE and anti-inflammatory.	[123]
	Fruit	Gallic acid, galloyl hexosides and ellagic acid derivatives.	Neuroinflammation.	Suppression of the gene expression of IL and inflammatory mediators (interferons and Tnfα).	Anti-inflammatory.	[124]
Figs *(Ficus carica)*	Pulp	Proanthocyanidins.	Memory, anxiety and learning in mice. Protein accumulation.	Enhancement of memory, spatial learning and motor coordination. Reduction in anxiety-related behavior through inhibition of the fibrillization of Aβ.	Anti-fibrillization.	[125]
Pomegranates, figs and dates	Pulp	Ferulic, sinapic, coumaric acids, luteolin, quercetin, catechin, epicatechin.	Neuroinflammation and protein accumulation in mice.	Suppression of Aβ levels. Delays in the formation of senile plaques. Reduction in pro-inflammatory ILs and TNF-α expression. Improvement in ATP formation.	Antiamyloidogenic and anti-inflammatory.	[30,126]
Olive and hibiscus *(Hibiscus sabdariffa)*	Leaf and Flower	Elenolic acid, oleuropein and hibiscus acid.	Apoptosis in Wistar rats (*in silico*).	Increase in cell viability under oxidative stress conditions. Reduction in mitochondria membrane potential loss, and reversion of caspases to basal levels. Reduced apoptosis mediated by oxidative stress.	Anti-MMP and antiapoptotic.	[41]
Apple *(Malus domestica)*	Pulp + Peel	Quercetin and dihydroxybenzoic acid.	Neural precursor cells in mice.	Promotion of cellular survival and neuronal differentiation. Presence of different pro-neurogenic compounds. Increase in neural precursor cell proliferation and neurogenesis. Inducement of endogenous antioxidants.	Pro-neurogenic and antioxidant.	[127]
Persimmon *(Diospyros kaki)*	Leaf	Myricetin, kaempferol, quercetin, hyperoside, astragalin and vitexin.	Neuroinflammation, antioxidant enzymes and apoptosis.	Decreased number of activated microglia and astrocytes reducing specific markers expression. Attenuation of NF-kB nuclear translocation. Improvement in SOD, GSH and CAT activity, and in PI3K and p-Akt levels.	Anti-inflammatory, antioxidant and anti-apoptotic.	[14]
Walnut *(Juglans regia)*	Kernel	Flavonoids, phenolic acids (ellagic), etc.	LPO, endogenous enzymes and mitochondrial dysfunctions in mice.	Reduction in LPO processes. activities. Improvement in SOD, CAT, GSH and GPx activity. Enhancement of dopamine levels inhibiting MAO enzymes. Restauration of mitochondrial complex-I activity.	Antioxidant and mitochondrial alleviator.	[59]
Black Pigweed *(Trianthema portulacastrum)*	Leaf	Protocatechuic, caffeic, chlorogenic, and ferulic acids.	Antioxidant potential and AChE inhibition in albino mice (*ex vivo*).	Reduction in TL and TRC. Significant increase in enzymatic antioxidants as well as hippocampal ACh levels.	Antioxidant and anti-AChE.	[68]
Hardy rubber-tree *(Eucommia ulmoides)*	Flower	All types of flavonoids. Anthocyanins and chalcones.	Protein accumulation and apoptosis. AChE inhibition (*ex vivo*).	Increase exercise capacity. Low number of Aβ plaques. Inhibition of AChE. Reduction in apoptotic cells. Regulate the expression of autophagy-related genes (ulk).	Anti-aAnti-autophagocytic.	[10]
	Leaf	Chlorogenic acid, quercetin, rutin, isoquercitrin, astragalin, etc.	Apoptosis in zebrafishes.	Inhibition of vasculature loss. Reduction in apoptotic cells. Relief of locomotor impairments. Upregulation of transcript levels of genes such as ulk2 and ulk1b.	Pro-autophagocytic.	[128]
Velvet bean *(Mucuna pruriens)*	Seed	Isoflavanones and alkaloids.	Antioxidant potential and apoptosis in albino mice.	Increase in neurotransmitter levels. Promotion of cell survival by increasing Akt activity and repairing OTA damage, mediating in neuronal apoptosis through PI3K/Akt signaling pathway.	Pro-synaptic and antiapoptotic.	[97]
Oat *(Avena sativa)*	Seedling	Feruloylquinic acid, isoorientin, vitexin, avenacosides A and B, diosgenoside, etc.	LPO, protein aggregation and neuroinflammation in mice (*ex vivo*).	Possible inhibition of LPS-induced ROS in microglia. Drastic reduction in numbers and size of Aβ plaques, and significant drop in BACE1 expression. Decrease in neuroinflammatory cells. Impact on MAPK signaling.	Anti-amyloidogenic, antioxidant and anti-inflammatory.	[98,99]
*Caesalpinia crista* *(Caesalpinia crista)*	Leaf	Gallic, coumaric and ferulic acids, rutin and genistin.	AChE inhibition, neuroinflammation, endogenous antioxidant system and LPO in rats.	AChE activity inhibition, interference at mRNA levels for the downregulation of pro-inflammatory cytokines and increase in NTF. Restoration of CAT, GSH and GST levels. Decrease in MDA levels.	Anti-AChE, anti-inflammatory and antioxidant.	[129]
Saffron *(Crocus sativus)*	Stigma	Carotenoids (crocin) and others.	Neuroinflammation and apoptosis in albino rats.	Decrease in caspase-3, COX-2 and GFAP expression, and MDA, TNF-α and IL6 levels.	Anti-apoptotic, anti-inflammatory and antioxidant.	[130]
Ginger *(Zingiber officinale)*	Rhizome	6-gingerol (phenolic).	Mitochondrial biogenesis in mice.	Mitochondrial biogenesis promotion, increase in mitochondrial mass, mtDNA copy number and ATP production. Regulation of AMPK-PGC1α pathway.	Mitochondrial alleviator.	[131]
*Guiera senegalensis* *(Guiera senegalensis)*	Leaf	Quercetin, glycosides, catechin, apigenin, epigallocatechin, caffeic, chlorogenic, cinnamic and ellagic acids.	Endogenous antioxidant system, LPO and AChE inhibition in *Danio rerio*.	Enhancement of antioxidant defense by preventing decrease in SOD, CAT, GPX and GSH, and suppressing the increase in MDA and protein peroxidation. AChE inhibition.	Antioxidant and anti-AChE.	[67]
Big-leaf mahogany *(Swietenia macrophylla)*	Seed	Alkaloids, terpenoids, tannins and flavonoids.	Downregulation of enzymes and cytokines in albino rats. Protein deposition.	Diminish inflammatory cascades reducing the neuronal effect of TNF-α. Significant improvement in GSH, SOD, CAT, MDA, NO and AChE levels. Avoidance of beta-amyloid depositions.	Anti-inflammatory, anti-AChE and anti-amyloidogenic.	[21]
Ming aralia *(Polyscias fruticose)*	Leaf	Proanthocyanidins, saponins, flavonoids, etc.	Parkinson on dUCH-knockdown *D. melanogaster*.	Improvements in flies’ mobility. Decrease in the degeneration of dopaminergic neurons.	Anti-PD.	[8]

DHA: Docosahexaenoic Acid; ARA: Arachidonic Acid; NTF: Neurotrophic Factor; Petr.: Petroleum; ULK1: Unc-51-Like Kinase 1; Bcl-2: B-cell lymphoma 2; GPx: Glutathione Peroxidase; Isoprop.: Isopropyl; BDNF: Brain-Derived Neurotrophic Factor; GR: Glutathione Reductase; OTA: Ochratoxin A; GST: Glutathione S-Transferase; AMPK: Adenosine Monophosphate-Activated Protein Kinase; PGC1α: Peroxisome Proliferator-Activated Receptor Gamma Coactivator 1-Alpha; TL: Transfer Latency; TRC: Time taken to Recognize the reward Chamber; DA: Dopaminergic.
